# Review of Homæopathy, Allopathy, and “Young Physic,” by L. W. Lawson, Prof. Trans. University

**Published:** 1846-10

**Authors:** 


*Review of Homœopathy, Allopathy, and "Young Physic," by L. W. Lawson, Prof. Trans. University.*—Prof. Lawson has reviewed at considerable length, the article by Dr. Forbes, of which so much has been said and written of late. His review originally appeared in the Western Lancet, of which Dr. L. is the editor. He has been induced, however, for the sake of giving it greater circulation, to publish it in a pamphlet form. That the publication of Dr. Forbes was in many respects injudicious, Dr. F., himself, would seem to acknowledge, since he has deemed it necessary to come out with an apology, and to correct various misconceptions to which his paper has given rise. The critique by Prof. Lawson, sets forth in a lucid manner the absurdities of Homœopathy, and illustrates the impro-propriety of some of the unwise concessions of Dr. Forbes.

